# AFB1 and OTA Promote Immune Toxicity in Human LymphoBlastic T Cells at Transcriptomic Level

**DOI:** 10.3390/foods12020259

**Published:** 2023-01-06

**Authors:** Massimo Frangiamone, Manuel Lozano, Alessandra Cimbalo, Guillermina Font, Lara Manyes

**Affiliations:** Laboratory of Food Chemistry and Toxicology, Faculty of Pharmacy, University of Valencia, 46100 Burjassot, Spain

**Keywords:** mycotoxins, Jurkat cells, RNA-sequencing, mechanism of action, immune toxicity

## Abstract

Aflatoxin B1 (AFB1) and ochratoxin A (OTA) are typical contaminants of food and feed, which have serious implications for human and animal health, even at low concentrations. Therefore, a transcriptomic study was carried out to analyze gene expression changes triggered by low doses of AFB1 and OTA (100 nM; 7 days), individually and combined, in human lymphoblastic T cells. RNA-sequencing analysis showed that AFB1-exposure resulted in 99 differential gene expressions (DEGs), while 77 DEGs were obtained in OTA-exposure and 3236 DEGs in the combined one. Overall, 16% of human genome expression was altered. Gene ontology analysis revealed, for all studied conditions, biological processes and molecular functions typically associated with the immune system. PathVisio analysis pointed to ataxia telangiectasia mutated signaling as the most significantly altered pathway in AFB1-exposure, glycolysis in OTA-exposure, and ferroptosis in the mixed condition (Z-score > 1.96; adjusted *p*-value ≤ 0.05). Thus, the results demonstrated the potential DNA damage caused by AFB1, the possible metabolic reprogramming promoted by OTA, and the plausible cell death with oxidative stress prompted by the mixed exposure. They may be considered viable mechanisms of action to promote immune toxicity in vitro.

## 1. Introduction

Aflatoxin B1 (AFB1) is the most toxic compound produced by several species of *Aspergillus,* which contamination occurs in a broad range of food commodities [1]. Once ingested, AFB1 is metabolized in the liver releasing reactive metabolites that are considered the causative agents of growth suppression, malnutrition, immune system alterations, and onset of hepatocellular carcinoma [2]. AFB1-toxicity has been also reported in the pancreas, bladder, kidney, and central nervous system [3,4]. In view of this, AFB1 is classified in group 1 as carcinogenic to humans by the International Agency for Research on Cancer (IARC) [5].

Another widespread mycotoxin, ochratoxin A (OTA), is mostly synthesized by *Penicillium verrucosum*, *Aspergillus ochraceus* and *Aspergillus niger*. Typical OTA-contaminated foods are cereals, wine, tea, coffee, cheese, meat, fruits, dried fruits, spices, and vegetables [6]. OTA is firstly absorbed in the kidney’s proximal tubule, and its slow excretion led to a potential accumulation in the body [7]. Several studies have demonstrated as OTA exposure can lead to hepatotoxicity, nephrotoxicity, and neurotoxicity [8,9]. According to this evidence, OTA has been classified in group 2B as a possible human carcinogen [5].

AFB1 and OTA are also well-known to be immune toxic agents in vitro and in vivo [10,11]. Exposure to AFB1 may induce an immune and pro-inflammatory response in macrophages with reactive oxygen species (ROS) generation, autophagy, and extracellular trap formation [12]. In macrophages, AFB1 also aggravated swine influenza virus infection, inflammation and damage of pulmonary tissue by activating TLR4-NFκB signaling pathway [13]. The suppression of immune response has been also observed in rats and broilers [14,15,16]. Similarly, OTA may disrupt the phagocytosis function of heterophils with intracellular ROS production [17]. In macrophages, OTA exposure was also correlated with rheumatoid arthritis progression [18], while in vivo OTA administration provoked apoptosis, immune stress, inflammation, and spleen damage [19]. Furthermore, it has been observed that AFB1 and OTA in combination caused inflammation, oxidative stress, and apoptosis in 3D4/21 cell line via NF-κB signaling pathway [20]. However, very little information on AFB1 and OTA-induced toxicity in human T lymphocytes has been reported, and their underlying mechanism has rarely been investigated.

In toxicological studies for human health risk assessment, the use of low experimental doses is necessarily required to reproduce a real scenario [21]. In human blood, AFB1 and OTA concentrations varied according to the studied population [22]. For AFB1, doses ranging from 0.6 to 237 nM were observed in the serum of Gambian children, while low values between 0.5 and 4 nM were detected in serum samples from healthy Iraqi patients [23,24]. As regards OTA, values of 22–25 nM were observed in plasma samples from Chinese and Bolivian patients, whereas only 2 nM in the serum of Swedish adolescents [25,26,27]. In relation to AFB1-metabolites, their detection is quite difficult [28]. For the main AFB1-metabolite, i.e., AFM1, extremely low values ranging from 0.01 to 0.03 nM were observed in urinary samples from Brazilian, Guinean, and Bangladeshi patients [24,29,30]. Regarding OTA-metabolism, the European Food Safety Authority (ESFA) reported that in vivo OTA-biotransformation, especially in humans and animals, is very low [31]. For OTα, the main OTA-metabolite, values of 0.5 nM were detected in the plasma of Bangladeshi adolescents as well as in urinary samples from Spanish and German patients [32,33,34]. Nevertheless, OTα has been described as a non-toxic compound and its formation has been considered an important OTA-detoxification pathway [31,35].

In light of the above, a transcriptomic analysis was designed to assess AFB1 and OTA toxicological effects at low doses (both at 100 nM), individually and in combination, after seven days of exposure the gene expression profile of Jurkat cells.

## 2. Material and Methods

### 2.1. Chemicals

The reagents and compounds used for cell culture, Roswell Park Memorial Institute (RPMI)-glutamax medium, fetal bovine serum (FBS), penicillin/streptomycin, and phosphate buffer saline were purchased by Sigma Chemical Co. (St. Louis, MO, USA); Dimethyl sulfoxide (DMSO) and methanol were obtained from Fisher Scientific (Madrid, Spain); Deionized water (<18, MΩcm resistivity) was obtained using Milli-QSP^®^ Reagent Water System (Millipore, Bedford, MA, USA); AFB1 (MW: 312.28 g/mol) and OTA (MW: 403.81 g/mol) standards were acquired from Sigma-Aldrich (St. Louis, MO, USA). Stock solutions were prepared in methanol solvent at a concentration of 1000 mg/L and kept at −20 °C.

### 2.2. Cell Culture and Exposure Conditions

Jurkat cells (ATCC-TIB152) were maintained in RPMI-glutamax medium complemented with 100 U/mL penicillin, 100 mg/mL streptomycin and 10% FBS. Cells were incubated at pH 7.4, 5% CO_2_ at 37 °C and air atmosphere at a constant humidity of 95%. The medium was changed every 2–3 days. To achieve the goal of the study, Jurkat cells were plated at a density of 2.5 × 10^5^ cells/mL in 6-well tissue culture plates and exposed for 7 days to DMSO solvent at 0.1% as control condition in maintenance medium (*n* = 3) as well as AFB1 and OTA at 100 nM, individually and in combination, in 0.1% DMSO (*n* = 3).

### 2.3. RNA Extraction and Next Generation Sequencing (NGS)

Firstly, RNA was extracted from Jurkat cells and purified from DNA contamination by using a ReliaPrep^TM^ RNA Cell Miniprep System Kit (Promega, WI, USA). Quantity and quality of obtained RNA were assessed using a NanoDrop™ 2000 spectrophotometer (Thermo Scientific™, Madrid, Spain), showing 260/280 nm and 260/230 nm ratios both around 2.

Secondly, Illumina NextSeq 500, supplied by the Genomics section of the Central Service for Experimental Research (SCSIE, University of Valencia), was employed for sequencing high-quality RNA samples, being the Integrity Numbers above 8. The standard protocol of Illumina was also carried out to create RNA-seq libraries, using TruSeq-stranded mRNA. Subsequently, results were generated as one archive for each sample, 12 in total.

### 2.4. Data Processing

FastQC software v0.11.8 (Babraham bioinformatics, Cambridge, UK) was used to calculate the percentage of mapped reads and to ensure quality control (QC) of reads [36]. The trimming was carried out employing FASTX-Toolkit v0.13 [37], eliminating bases from 5′- and 3′-extremes. Then, reads with low quality and identical sequences were reduced into a single sequence but maintained counts. The trimmed reads alignment was performed by Spliced Transcripts Alignment to a Reference (STAR) software v2.7 (Cold Spring Harbor Laboratory, New York, NY, USA) [38], using the Genome Reference Consortium Human Build 38 version as a reference. SAM tools software v1.10 (GitHub, San Francisco, CA, USA) [39] allowed to transform Sequence Alignment Map (SAM) files into their binary version (BAM). BAM files were used with STAR to generate an expression matrix in *R* software [40]. Annotation, normalization and statistical analysis were performed according to the user guide of *edgeR* package [41] to contrast differential expression among mycotoxin exposures and control, using gene-wise negative binomial generalized linear models with quasi-likelihood tests. Differentially expressed genes (DEGs) with *p*-value ≤ 0.05 were considered significant. Venn diagrams and heat maps were built by using *Vennerable* and *pheatmap* packages [42,43] to assess coincident DEGs between conditions [44,45].

### 2.5. DEGs Analysis

DEGs were submitted to gene ontology (GO) analysis by ConsensusPathDB [46]. Pathway assignments were carried out through PathVisio software (University of Maastricht, Maastricht, The Netherlands) by using WikiPathways as biological pathways database [47,48]. Z-score > 1.96 and *adjusted p* ≤ 0.05 were used as thresholds to identify significant pathways.

### 2.6. Gene Selection and Primer Design

Specific primers for each gene were designed by Primer-BLAST establishing the default software settings with PCR products of amplification ranging from 97 to 145 bp and melting temperature of 60 °C. qPCR analysis was performed by StepOne Plus Real-time PCR instrument (Applied Biosystems, Foster City, CA, USA). The reliability of primer amplification was determined from standard curves of each gene, by measuring linearity (R^2^ values) and efficiency of Ct mean values against the log cDNA dilution factor. Table 1 reports primers employed in the present study.

### 2.7. Reverse Transcription and qPCR

Amplification solutions were prepared in 96 well plates using SYBR Green as fluorescent dye. Reactions mixes consisted of 100 ng template, 500 nM of each primer, and the required amount of 2× Fast SYBR Green in a reaction volume of 10 μL. The PCR temperature cycling conditions for cystatin A (CSTA) and DNA nucleotidylexotransferase (DNTT) were as follows: initial denaturation step at 95 °C for 10 min to activate Taq DNA polymerase, followed by 40 cycles of denaturation at 95 °C for 15 s, annealing at 58 °C for 30 s, and elongation at 72 °C for 15 s. The melting curve was generated by heating the amplicon from 60 to 90 °C. Threshold cycles (Ct) were generated by StepOne Plus Software version 2.3 and relative gene expressions were assessed using the 2^−ΔΔCT^ method [49]. The relative quantification values were transformed to log2 (Log2RQ) for normalization. Samples were run in triplicate according to Minimum Information for Publication of Quantitative Real-Time PCR Experiments guidelines [50].

### 2.8. Statistical Analysis

Statistical analysis was carried out by ΔCt values (experimental Ct and housekeeping Ct mean) obtained using the StepOne Plus Software version 2.3 (Applied Biosystems, Foster City, CA, USA). Variances among groups were evaluated by Levene’s test and all the group variances were equal. T-Student defined differences between controls and treated groups. A *p* value ≤ 0.05 was considered for statistically significant differences.

## 3. Results

### 3.1. DEGs Profile

**Figure 1:**
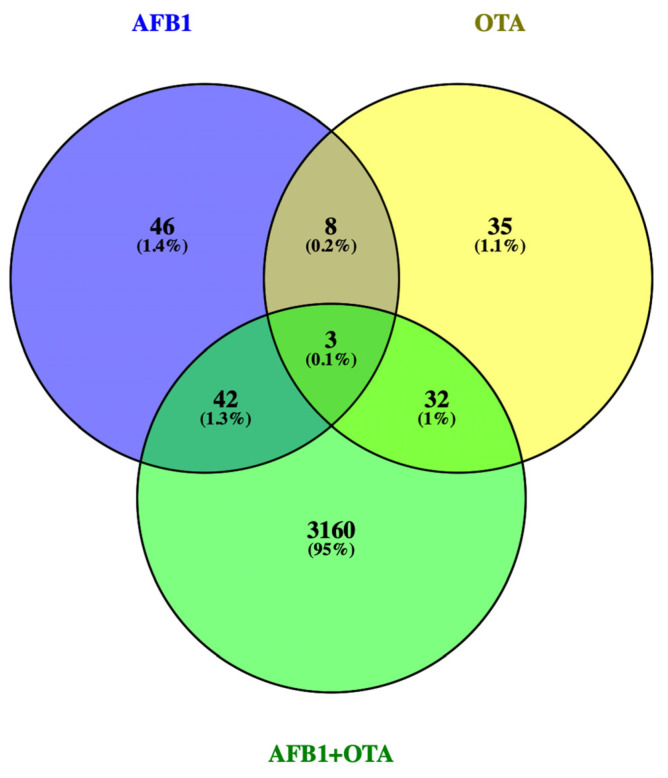
Venn diagram for the DEGs found by RNA-seq in Jurkat cells treated with AFB1, OTA, AFB1 + OTA at 100 nM in 0.1% DMSO compared to 0.1% DMSO control exposure. DEGs *p*-value ≤ 0.05 and log2FC |0.5|.

Gene expression of lymphocytes T exposed for 7 days to AFB1 and OTA (100 nM), individually or in combination, significantly differs from the expression of untreated cells. Considering a *p*-value ≤ 0.05 and log2FC |0.5|, AFB1-exposure resulted in 99 DEGs with 61 up- and 38 down-regulated. A total of 77 DEGs (23 up- and 54 down-regulated) were obtained in OTA exposure and 3236 DEGs (2255 up- and 981 down-regulated) in the combined AFB1-OTA exposure. Overall, the total DEGs number was 3412 with 68.5% up-regulation and 31.5% down-regulation by compromising the usual expression levels up to 16% of the human genome, containing 21,000 protein-coding genes (HGNC database). Interestingly, 42 DEGs (1.3%) overlapped in AFB1 and combined exposure, 8 DEGs (0.2%) among AFB1 and OTA individually, and 32 DEGs (1%) between OTA and mixed exposure. Solely 3 DEGs (0.1%): DNTT, adhesion G-protein-coupled receptor E1 (ADGRE1), and voltage-dependent T-type calcium channel subunit alpha-1I (CACNA1l) were found by overlapping all conditions (Figure 1). Although DNTT was up-regulated for each exposure, ADGRE1 and CACNA1l were down-regulated in AFB1 and OTA individually and over-expressed in the combined exposure.

### 3.2. GO and Pathway Identification

The characterization of biological processes (BP) and molecular functions (MF) in which DEGs are involved is a major step in this transcriptomics study. It includes the comparison between the DEGs list and the rest of the genome for over-represented functions and gene set enrichment analysis [51,52]. Table 2 shows relevant GOs with their relative categories obtained by the over-representation analysis in ConsensusPathDB.

#### 3.2.1. AFB1-Exposure

Regarding the AFB1 condition, the over-representation analysis by ConsesusPathDB provided a list of GO terms in which several BP linked to system development, cell adhesion, and regulation of the immune system process were some of the most over-represented. MF associated with cation, steroid, and GTPase binding, DNA and RNA polymerase transcription factor activity, and cytokine receptor function were significant in the DEGs set at levels 3 and 4. Plasma membrane for cellular components (CC), was significantly represented in the individual AFB1-exposure (Table 2).

PathVisio analysis reported a number of 3691 data points (N), among them 753 met criterion (R). (N) denotes the total number of genes measured in the dataset where (R) indicates the filter analysis criterion [47]. Several pathways were statistically significant after AFB1 exposure (Z-score > 1.96; *adjusted p*-value ≤ 0.05). According to the number of genes affected, the most significant pathways were as follows: ApoE and miRNA-146 in inflammation and atherosclerosis (80%), severe acute respiratory syndrome coronavirus 2 (SARS-CoV-2) actives NLRP3 inflammasome (67%), miRNA-124 interaction with cell cycle and differentiation (67%), and SARS-CoV-2 antagonizes innate immune activation (62%). The analysis also showed a significant alteration in the mitochondrial immune response to SARS-CoV-2 and ataxia telangiectasia mutated (ATM) signaling in development and disease, with 45% of genes affected in each pathway (Table 3).

**Figure 2:**
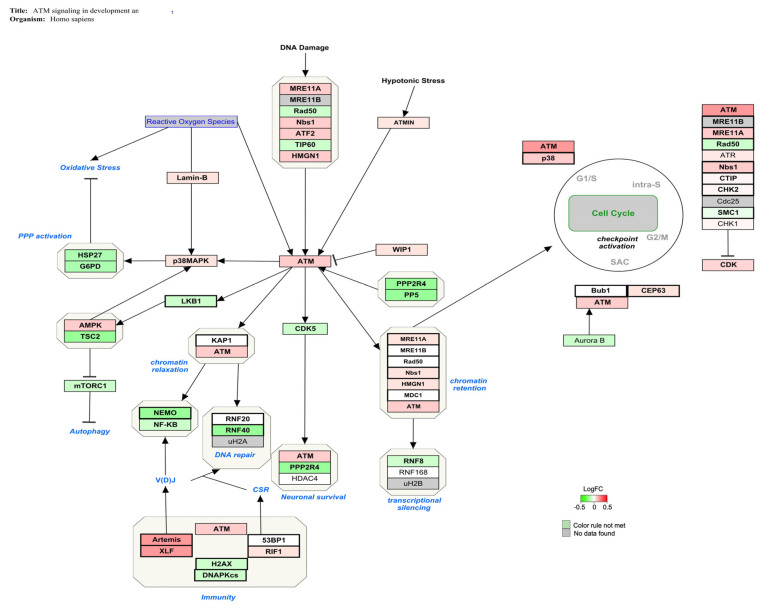
Genes involved in ATM signaling for *Homo sapiens* are shown in red and green. The up-regulated genes are represented in red and the down-regulated ones after AFB1 exposure (100 nM) are represented in green.

The genes involved in the ATM signaling pathway (Z-score: 4.15) for *Homo sapiens* are shown in Figure 2, indicating in red the over-expressed genes and in green the down-regulated genes ones. A slight alteration in ATM-related gene expression was obtained.

#### 3.2.2. OTA Exposure

The consensusPathDB analysis pointed to blood coagulation, secretion, hypoxia, hemostasis, and regulation of the immune and inflammatory response as some of the most over-represented BP upon OTA exposure. The activity of dioxygenase and ubiquitin proteins was the most significant MF in the DEGs set at levels 3 and 4, whereas CC such as basolateral plasma membrane and its integral components were significantly affected (Table 2).

PathVisio analysis reported a total number of 3662 data points, of which only 193 met the criterion. Several pathways were statistically significant upon OTA exposure (Z-score > 1.96; *adjusted p*-value ≤ 0.05). Based on the number of genes affected, the most significant pathways were glycolysis (43%) and the Cori cycle (34%). The analysis indicated a significant alteration in HIF1α and PPAR-γ regulated glycolysis (29%) and innate immune response to SARS-CoV-2 with 25% of genes affected (Table 4).

**Figure 3:**
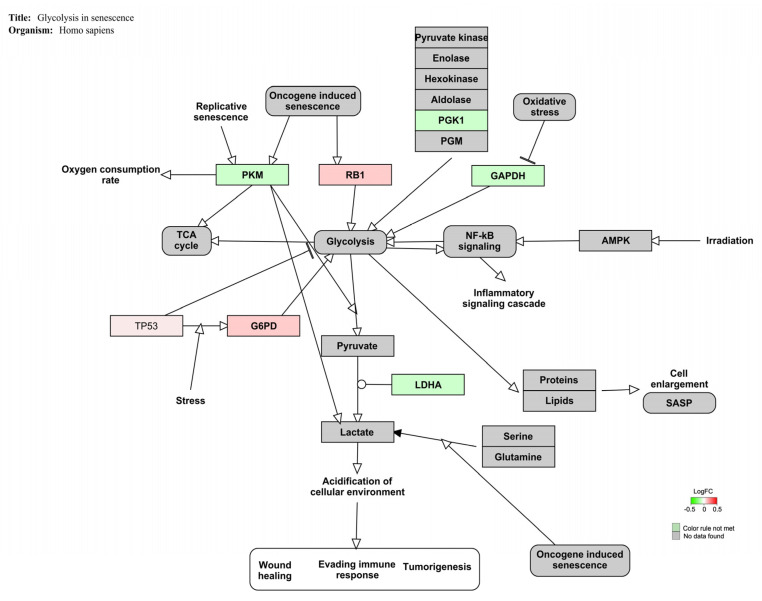
Genes implicated in glycolysis senescence pathway for *Homo sapiens* are represented in red and green. The up-regulated genes are represented in red and the down-regulated ones upon OTA exposure (100 nM) are represented in green.

Figure 3 displays the genes involved in the glycolysis signaling pathway (Z-score: 4.45) for *Homo sapiens*. In this case, low values of fold changes were observed.

#### 3.2.3. AFB1-OTA Combined Exposure

The analysis conducted by ConsensusPathDB showed that in AFB1-OTA mixed exposure, the most over-represented BP were the regulation of immune system process, cytokine production, cell adhesion, lymphocyte differentiation, and T cell cytotoxicity, among others. MF linked to G protein, cytokine receptor activity and C-C chemokine binding were statistically significant in the DEGs set at levels 4 and 5. CC such as cell cortex and membrane, chromatin, blood microparticle, and MHC class I protein complex were significantly affected to a greater extent (Table 2).

PathVisio analysis showed 3744 data points, of which a large number (2489) matched the criterion. Solely 9 of the pathways were statistically relevant following the combined exposure (Z-score > 1.96; *adjusted p*-value ≤ 0.05). In this case, all pathways found showed more than 84% of genes affected. The 100% of genes involved in the tricarboxylic acid cycle (TCA), dual hijack model of HIV infection, and hyperlipidemia were statistically altered (Table 5).

**Figure 4:**
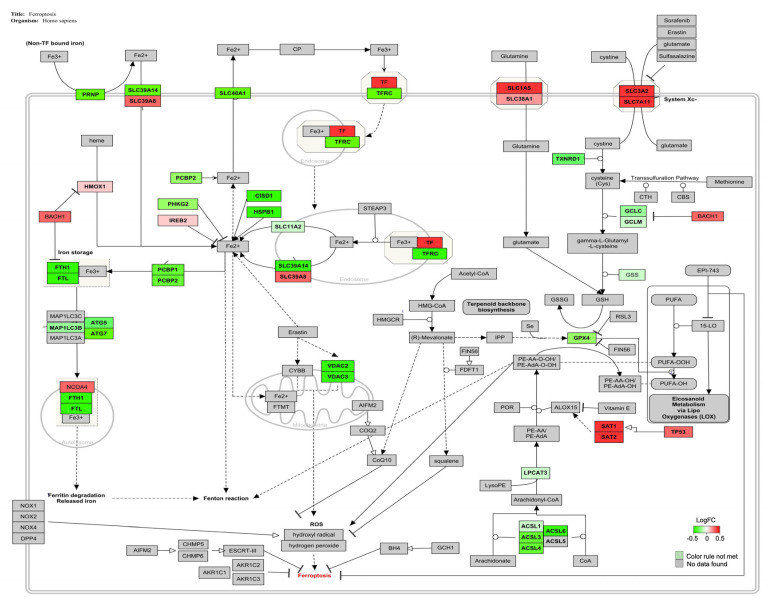
Genes involved in ferroptosis pathway for *Homo sapiens* are represented in red and green. The up-regulated genes are represented in red and the down-regulated ones following AFB1-OTA exposure (100 nM for both toxins) are represented in green.

The genes involved in the ferroptosis signaling pathway (Z-score: 2.41) for *Homo sapiens* are shown in Figure 4, and the majority of them were down-regulated.

### 3.3. Validation of NGS Results by qPCR

**Figure 5:**
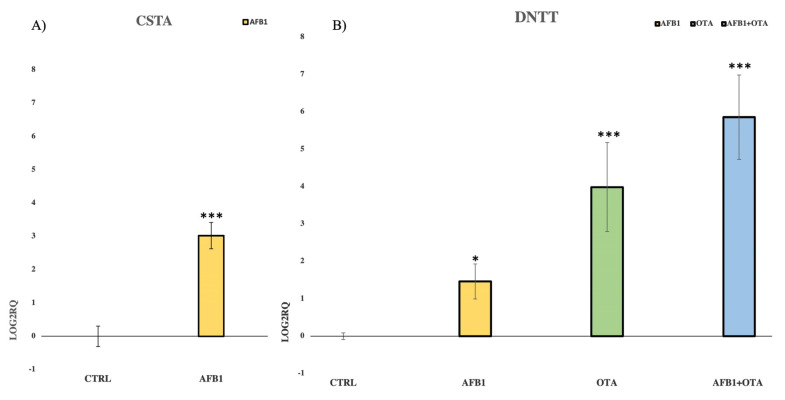
Bar plots showing the relative expression of (**A**) CSTA and (**B**) DNTT when compared to the control (Log2RQ = 0) after 7 days of exposure in Jurkat cells to AFB1 (100 nM) for CSTA and to AFB1, OTA, AFB1 + OTA (100 nM in all cases) for DNTT by qPCR. RQ: relative quantification; CTRL: control; CSTA: cystatin A; DNTT: DNA nucleotidylexotransferase. *p* ≤ 0.05 (*), *p* ≤ 0.001 (***).

The expression of CSTA, a cysteine protease inhibitor gene, was assessed after exposure to AFB1 whereas DNTT expression, an independent DNA polymerase gene, was evaluated upon exposure to AFB1, OTA, and mycotoxins mixture. The experimental conditions used in the qPCR analysis were the same as those used for the NGS assay. Additionally, CSTA and DNTT were chosen because they were the most affected genes in the sequencing analysis. Moreover, qPCR assay confirmed NGS results with the strong up-regulation of CSTA and DNTT when compared to the control (Figure 5A,B). A ribosomal protein 18S was employed as endogenous gene control.

## 4. Discussion

In the present study, the possible mechanism of action (MoA) by which low doses of AFB1 and OTA, comparable with those found in human blood, promoted immune toxicity was investigated after seven days in vitro exposure. The choice of this exposure time reflects the peak of the immune response *in vivo*, which precisely occurs after the seventh day of infection. Thereafter, the number of T lymphocytes undergoes a programmed contraction as well as the immune system gradually tends to deactivate [53,54]. Reproducing a realistic scenario, it is interesting to observe as the number of DEGs was significantly increased in the combined exposure (3236) when compared to the individual ones (AFB1 DEGs = 99 and OTA DEGs = 77). This finding is relevant to human health risk assessment, as the human population is constantly exposed to multiple mycotoxins contaminating food and our findings may suggest that AFB1 and OTA had an additive in vitro effect [22]. Similar results were obtained by [55], exposing HepG2 liver cells with high concentrations of AFB1 (1.5–150 μM; 3 and 24 h) and OTA (50–800 μM; 3 and 24 h). It has been observed more prominent toxic effects in the combined exposure compared to individual ones, suggesting a synergism between mycotoxins. In Vero kidney cells, AFB1 and OTA (1–50 μM; 24 h) not only showed an additive cytotoxic effect but also synergism to promote genotoxicity with increased DNA fragmentation [56]. It has been also shown that OTA significantly increased AFB1 mutagenicity with a higher percentage of mutations than AFB1 alone, indicating the potential risk of mycotoxins co-occurrence [57]. On the contrary, different findings were obtained by [58], treating chicken LMH liver cells with AFB1 (0–3 μM; 48 h) and OTA (0–20 μM; 48 h). The cytotoxicity assay and transcriptome analysis revealed the antagonist effect between mycotoxins to induce chicken liver toxicity. The discrepancy in results was probably associated with the use of experimental conditions (doses, exposure time, and non-human cell line) less realistic compared to those employed in the present study.

### 4.1. AFB1 Exposure

ATM signaling was the most affected pathway after AFB1 exposure (Table 3). It is well known that ATM plays a major role in the cellular response to DNA damage, by involving ATM protein kinase as the main downstream signal effector [59]. Several studies showed that in vitro and in vivo exposure to AFB1-induced DNA double-strand breaks (DSBs), activation of ATM signaling, and the up-regulation of all ATM kinases related to chromatin relaxation, cell cycle regulation, and immunity [60,61,62]. It has been also demonstrated that low AFB1 doses (5–80 nM; 24 h) were sufficient to promote DNA damage and ATM up-regulation in BEAS-2B cells with possible genome alterations in the human respiratory system [63].

ATM signaling is also a core component of the DNA repair system by activating the ubiquitin ligase RNF40, which in turn covers a key role in chromatin reorganization and timely DNA-DSB repair [64,65]. The reduced expression of RNF40 was associated with replicative stress, chromosomal instability, cell cycle checkpoint inactivation, and inhibition of DNA repair mechanism in vitro [66,67,68]. In turkey embryos, exposure to AFB1 (1 μg/for injection; 24 h) has been shown to downregulate the activity of ubiquitin ligase and impair the cellular response to genotoxic damage [69,70]. Therefore, the activation of ATM signaling with up-regulation of ATM kinases and RNF40 down-regulation pointed to the induction of DNA damage and the disruption of the repair mechanism as a possible MoA by which AFB1 induces immune toxicity in vitro (Figure 2).

The central position of ATM signaling to maintain genomic stability is demonstrated by its involvement in the G_2_/M cell cycle transition [71]. It has been observed as AFB1-exposure prompted the up-regulation of ATM kinase, checkpoint kinase 2 (chk2) and Ataxia telangiectasia Rad3-related protein (ATR), which in turn caused G_2_/M cell cycle arrest in bronchial epithelial cells [63]. Similar findings were also observed in the chicken bursa of Fabricius, spleen, and jejunum, thus confirming the correlation between in vivo AFB1 administration and the disruption of cell cycle machinery at G_2_/M stage by ATM signaling activation [62,72,73]. In line with these findings, 7 days of exposure to AFB1 can induce a potential alteration in cell cycle distribution (Figure 2). However, gene expression changes (i.e., ATR, chk2) are very mild and further research is needed to confirm this hypothesis.

Moreover, ATM signaling is involved in T cell development by controlling V(D)J recombination process, which is indispensable to constitute the variable domain of T cell receptor [74,75]. Alterations in ATM signaling and its main effectors can disrupt V(D)J process and predispose human T cells to leukemia and lymphoma [76]. In this context, Artemis protein, a well-known ATM substrate, has a critical role in V(D)J recombination [77,78]. It has been also reported the association between in vitro deleterious effects such as immunodeficiency, cell cycle arrest, DNA damage, and Artemis over-expression [79,80,81]. Thus, ATM signaling activation with ATM kinase and Artemis upregulation may suggest the possible interference of AFB1 in T cell recombination and functionality (Figure 2).

### 4.2. OTA Exposure

Mycotoxins can induce in vitro toxicity by reprogramming cell metabolism [82]. For instance, OTA (1.25–5 μM; 24 h) promoted mitochondrial toxicity by reprogramming energy metabolism from oxidative phosphorylation (OxPhos) to glycolysis in human gastric cells [83]. Similar metabolic disturbances were obtained by [84] in human esophageal cells after 24 h exposure to OTA (2.5–10 μM; 24 h) and by [85], analyzing the liver of rats treated with moderate doses of AFB1 in contaminated feed (1.6 mg/kg; 12 days). In line with this evidence, PathVisio analysis revealed the promotion of metabolic reprogramming and pointed to glycolysis as the most affected pathway after 7 days of in vitro exposure to OTA (Table 4).

Figure 3 showed that all genes related to the glycolysis pathway were slightly affected by mycotoxins exposure. In detail, retinoblastoma 1 (RB1) is a tumor suppressor involved in cell cycle regulation, cell differentiation, proliferation, and death [86]. It has been demonstrated that RB1 over-expression was associated with metabolic changes and glycolysis promotion in human breast cells [87]. Similarly, the up-regulation of glucose-6-phosphate dehydrogenase (G6PD), a pivotal enzyme in NADPH production and cell redox balance, enhanced glycolysis activation in acute myeloid leukemia cells [88]. G6PD over-expression has been also observed in beauvericin and enniatin, B promoted mitochondrial disturbance and reduced ATP production in vitro [89]. Phosphoglycerate kinase 1 (PGK1) catalyzes the first reaction of anaerobic glycolysis. The microarray analysis performed in HepG2 cells after OTA exposure (2.5–10 μM; 24 h) showed the downregulation of PGK1 and its involvement in OTA-disrupted liver metabolism [90]. Likewise, the reduced expression and activity of pyruvate kinase M2 (PKM2), which converts phosphoenolpyruvate to pyruvate with ATP production, has been correlated with OTA-induced metabolic disturbance and glycolysis in human gastric cells [83,91]. Additionally, glyceraldehyde-3-phosphate dehydrogenase (GAPDH), previously considered a simple housekeeping gene, has been shown to be involved in many cellular processes such as glycolysis and cell metabolism [92]. Indeed, GAPDH down-regulation was related to significant alterations in energy metabolism after exposure to ZEA (10 μM; 24 h) in human breast MCF10F cells [93]. Lastly, lactate dehydrogenase A (LDHA) catalyzes the last step of anaerobic glycolysis by converting pyruvate to lactate [94]. Interestingly, Fusaric acid (4 and 256 μM; 6 h), a neglected foodborne mycotoxin, downregulated LDHA gene expression, among others, and induced bioenergetic adaptations by switching energy metabolism from mitochondrial OxPhos to glycolysis in HepG2 cells [95]. The metabolic disturbance promoted by OTA exposure in Jurkat cells is further supported by the alteration of HIF1α, PPAR-γ, and Cori cycle signaling pathways (Table 4). HIF1a and PPAR-γ pathways are key regulators of metabolic reprogramming and glycolysis in vitro whereas the Cori cycle is a typical metabolic route activated with high lactate concentrations [96,97,98].

### 4.3. AFB1-OTA Combined Exposure

Ferroptosis was the main signaling pathway altered by the combined AFB1-OTA exposure with 85% of genes affected (Table 5). Ferroptosis is a type of regulated necrosis triggered by the combination of excessive intracellular iron overload, induction of lipid peroxidation with plasma membrane damage and inhibition of glutathione peroxidase 4 (GPX4) activity [99].

The intracellular iron accumulation can be mediated by several factors. Firstly, transferrin (TF), a strong inducer of the process, is a carrier protein that binds extracellular ferric iron (Fe^3+^) and transports it into cells through its transporter (TFR) by endocytosis [100]. It has been demonstrated the strong correlation between TF over-expression and ferroptosis induction in broiler hearts upon AFB1 administration in contaminated feed (1 mg/kg; 21 days). The down-regulation of Heat Shock Protein Family B1 (HSPB1), a negative regulator of intracellular iron uptake and accumulation, also confirmed the imbalance of iron levels in AFB1-promoted ferroptosis [101]. Conversely, TFR1 downregulation may lead to iron accumulation, oxidative stress, and ROS generation in vitro [102]. Indeed, RNA-seq analysis performed on DON-treated IPEC-J2 cells (0.5 μg/mL; 48 h) revealed the alteration of several genes related to iron homeostasis, including the down-regulation of TFR1, which was considered a key initial signal of ferroptosis [103]. Another pivotal driver of ferroptosis is the transcription factor BTB domain and CNC homolog 1 (BACH1), a regulator of iron metabolism. In vitro upregulation of BACH1 can induce the repression of several genes involved in iron storage (such as ferritin, a protein complex consisting of heavy, FTH1, and light chains, FTL), thereby causing the release of unstable iron into the cytoplasm and worsening the ferroptotic condition [104,105].

High concentrations of free iron in the cytoplasm can increase the production of endogenous hydrogen peroxide and hydroxyl radical by the Fenton reaction, by promoting lipid peroxidation [106,107]. During ferroptosis, lipid oxidation occurs as an intermediate event, in which ASCL4 and ASCL3 are the main regulators [108,109]. For instance, T-2 toxin (2.5–10 nM for 6–20 h) promoted ferroptosis, lipid peroxidation, and intracellular ROS generation through the alteration of several genes expression, including the slight downregulation of ASCL4 [110]. Likewise, the downregulation of ASCL3 has been related to ferroptosis and oxidative stress in vitro and in vivo [111].

GPX4, a selenium-dependent glutathione peroxidase, protects cells from lipid hydroperoxides formed during oxidative stress, using reduced glutathione as an enzyme co-substrate [112]. In human T cells, the reduced GPX4 activity can lead to lipid peroxidation and ferroptotic cell death, altered cellular homeostasis, and increased susceptibility to acute infections [99,113]. It has been also reported that exposure to AFB1 and OTA downregulated GPX4 gene expression inducing lipid peroxidation, ROS generation, and ferroptosis in several in vitro and in vivo models [114,115,116]. Based on this evidence, AFB1-OTA combined exposure may induce ferroptosis in Jurkat cells by altering the expression of several key genes involved in intracellular iron overload (TF, TFR1, BACH1, FTH1, FTL, and NCO4), lipid peroxidation (ASCL4, ASCL3, SAT1) and antioxidant cell defense (GPX4) (Figure 5). Interestingly, AFB1 and OTA also altered the PPAR-α signaling pathway, a novel route associated with ferroptosis due to its active function in lipid remodeling and peroxidation (Table 5) [117].

In the present study, AFB1 and OTA, individually and in combination, resulted in DNTT over-expression. Moreover, qPCR analysis confirmed not only NGS results but also the synergism between mycotoxins, as their combined transcriptional effect on DNTT was more pronounced than individual exposures. It is well known that DNTT plays a key role in T cell recombination and proliferation, showing a strong anti-apoptotic function. Consequently, DNTT up-regulation has been reported to confer resistance to tumor cells against chemotherapeutic agents [118]. Since the increased DNTT activity has been also found in peripheral blood cells derived from patients with acute lymphoblastic leukemia, it cannot be excluded as exposure to AFB1 and OTA may worsen the leukemic condition [119,120,121].

## 5. Conclusions

These results contribute to a better understanding of the main cellular pathways involved in AFB1 and OTA-induced immune toxicity in Jurkat cells. RNA-seq analysis revealed that ATM signaling pathway was the most altered in AFB1 exposure. In detail, potential alterations in G_2_/M cell cycle checkpoint, T cell recombination, and induction of DNA damage with impairment of repair mechanisms have been mainly related to AFB1 toxicity. Regarding OTA exposure, the glycolysis signaling pathway was the most affected. Therefore, energy metabolism reprogramming by glycolysis activation may be considered the main OTA-MoA in Jurkat cells. In combination, AFB1 and OTA mainly affected the ferroptosis signaling pathway. In this case, the potential accumulation of intracellular iron with lipid peroxidation and oxidative stress accompanied by the disruption of antioxidant cell defense may explain the intrinsic MoA by which the mixture of mycotoxins promoted immune toxicity in vitro. Although the transcriptome analysis identified the main pathways altered by low doses of AFB1 and OTA in a human T cell line, further investigations are required to confirm these hypotheses and to better explore the underlying mechanism by which AFB1 and OTA may weaken the immune system, by rendering it more susceptible to infections.

## Figures and Tables

**Table 1:** Gene names, forward (FS) and reverse (RS) primer sequences, PCR efficiency (E%) and R^2^ value for the target genes and the reference gene 18S rRNA.

Gene	FS	RS	E%	R^2^
18S rRNA	CGGCTACCACATCCAAGGAA	GCTGGAATTACCGCGGCT	105	0.990
CSTA	AAACCCGCCACTCCAGAAAT	GCACAGCTTCCAATTTTCCGT	107	0.991
DNTT	GCCTCGTCAAAGAGTGGACA	GTCTCTCTCAAACCGGGAGC	109	0.991

**Table 2:** Relevant GOs resulting from the over-representation analysis of the selected DEGs obtained by AFB1, OTA, AFB1 + OTA exposures (100 nM in all cases) in ConsensusPathDB.

Gene Ontology Term	Categories	Set Size	Genes Candidates	*p*-Value
AFB1 exposure				
GO:0098609 Cell-cell adhesion	BP 3	871	7 (0.8%)	<0.05
GO:0048731 System development	BP 3	4796	25 (0.5%)	<0.01
GO:0002684 Regulation of immune system process	BP 4	1238	9 (0.7%)	<0.05
GO:0004896 Cytokine receptor activity	MF 4	97	3 (3.1%)	<0.01
GO:0043169 Cation binding	MF 3	4346	22 (0.5%)	<0.05
GO:0051020 GTPase binding	MF 4	388	4 (1.0%)	<0.05
GO:0000981 DNA and RNA transcription activity	MF 3	1401	9 (0.6%)	<0.05
GO:0005496 Steroid binding	MF 3	100	2 (2.0%)	<0.05
GO:0035579 Plasma membrane	CC 4	91	2 (2.2%)	<0.05
OTA exposure				
GO:0046903 Secretion	BP 4	1469	12 (0.8%)	<0.001
GO:0007596 Blood coagulation	BP 3	344	5 (1.5%)	<0.01
GO:0007599 Hemostasis	BP 4	348	5 (1.4%)	<0.01
GO:0006954 Inflammatory response	BP 4	842	7 (0.8%)	<0.05
GO:0002683 Regulation of immune system process	BP 4	468	5 (1.1%)	<0.05
GO:0001666 Response to hypoxia	BP 3	338	4 (1.2%)	<0.05
GO:0051213 Dioxygenase activity	MF 3	90	2 (2.2%)	<0.05
GO:0004842 Ubiquitin-protein transferase activity	MF 4	431	4 (0.9%)	<0.05
GO:0016323 Basolateral plasma membrane	CC 3	242	3 (1.2%)	<0.05
GO:0016021 Integral component of membrane	CC 3	5401	22 (0.4%)	<0.05
AFB1-OTA exposure				
GO:0007155 Cell adhesion	BP 2	1457	48 (3.3%)	<0.001
GO:0001816 Cytokine production	BP 2	746	20 (2.7%)	<0.01
GO:0030098 Lymphocyte differentiation	BP 4	355	12 (3.4%)	<0.01
GO:0002684 Regulation of immune system process	BP 4	1238	30 (2.4%)	<0.01
GO:0001913 T cell cytotoxicity	BP 3	50	4 (8.0%)	<0.01
GO:0008528 G-protein-coupled peptide receptor activity	MF 4	150	8 (5.3%)	<0.01
GO:0004896 Cytokine receptor activity	MF 4	97	9 (9.3%)	<0.001
GO:0019957 C-C chemokine binding	MF 5	24	3 (12.5%)	<0.01
GO:0042612 MHC class I protein complex	CC 5	10	2 (20.0%)	<0.01
GO:0072562 Blood microparticle	CC 2	148	7 (4.7%)	<0.01
GO:0005938 Cell cortex	CC 5	304	11 (3.6%)	<0.01
GO:0000785 Chromatin	CC 4	1231	30 (2.4%)	<0.01

**Table 3:** Pathways overlapped in AFB1-exposure (100 nM) by PathVisio.

Pathway	Positive (r)	Measured (*n*)	Total	%	Z Score	*Adj. p*-Value
ATM signaling in development and disease	20	44	49	45	4.15	<0.01
ApoE and miR-146 in inflammation and atherosclerosis	4	5	13	80	3.31	<0.01
SARS-CoV-2 antagonizes innate immune activation	5	8	15	62	2.96	<0.01
Mir-124 predicts cell cycle and differentiation	4	6	8	67	2.81	<0.01
Mitochondrial immune response to SARS-CoV-2	8	18	62	44	2.54	<0.05
Activation of NLRP3 inflammasome by SARS-CoV-2	2	3	10	67	1.99	<0.05

**Table 4:** Pathways overlapped in OTA exposure (100 nM) by PathVisio.

Pathway	Positive (r)	Measured (*n*)	Total	%	Z Score	*Adj. p*-Value
Glycolysis in senescence	3	7	26	43	4.45	<0.01
Cori cycle	4	12	53	33	4.36	<0.01
Metabolic reprogramming	7	40	80	17	3.48	<0.01
HIF1α and PPAR-γ regulation of glycolysis	2	7	19	29	2.76	<0.05
SARS-CoV-2 antagonizes innate immune activation	2	8	15	25	2.5	<0.05

**Table 5:** Pathways overlapped in AFB1-OTA-exposure (100 nM) by PathVisio.

Pathway	Positive (r)	Measured (*n*)	Total	%	Z Score	*Adj. p*-Value
Ferroptosis	33	39	88	84	2.41	<0.05
PPAR-α pathway	14	15	28	93	2.21	<0.05
TCA cycle in senescence	9	9	30	100	2.13	<0.05
Dual hijack model of HIV infection	8	8	10	100	2.01	<0.01
Familial hyperlipidemia type 1	8	8	35	100	2.01	<0.05

## Data Availability

All related data and methods are presented in this paper. Additional inquiries should be addressed to the corresponding author.
